# Quantitative MRI reveals infrapatellar fat pad changes after running a marathon

**DOI:** 10.7717/peerj.19123

**Published:** 2025-03-20

**Authors:** Xiang Bo Zhao, Haifeng Zhao, Wen Juan Du, Hao Zhang

**Affiliations:** 1 The First School of Clinical Medicine, Lanzhou University, Lanzhou, Gansu, China; 2 The First Hospital of Lanzhou University, Lanzhou, Gansu, China; 3 Gansu Intelligent Imaging Medical Engineering Research Center, Lanzhou, Gansu, China

**Keywords:** Infrapatellar fat pad, Marathon running, Magnetic resonance imaging, MAGiC, Sports medicine

## Abstract

**Background:**

Marathon running, while offering health benefits, is associated with a high incidence of knee injuries. The infrapatellar fat pad (IFP) plays a critical role in knee joint homeostasis and injury mitigation. This study investigated IFP adaptations to the acute stress of marathon running using quantitative magnetic resonance imaging (MRI).

**Methods:**

Fourteen amateur marathon runners (12 male, two female) were prospectively enrolled and underwent 3.0T MRI (GE SIGNA Architect) one week before and after the marathon. Left knee imaging included MAGiC and IDEAL-IQ sequences. MAGiC sequences provide T1, T2, and proton density (PD) maps. IDEAL-IQ sequences yield fat fraction (FF), representing the relative amount of fat within the IFP, and the transverse relaxation rate (R2*) within the IFP. IFP volume and maximum cross-sectional area were quantified. Two experienced radiologists independently analyzed the images using dedicated software. Inter-observer reliability for quantitative MRI measurements was assessed using intraclass correlation coefficients (ICCs). Paired t-tests were used to compare pre- and post-marathon measurements of T1, T2, FF, R2* values, IFP volume and maximum cross-sectional area. Pearson correlation analysis explored relationships between changes in IFP parameter changes and participant characteristics. *P* < 0.05 was considered statistically significant for all analyses.

**Results:**

Post-marathon, IFP FF significantly increased (*p* < 0.05), while IFP volume significantly decreased (*p* < 0.05), T1 and T2 values showed a decreasing trend. IFP appeared morphologically compressed post-marathon. A significant negative correlation was found between FF change and both body weight and body mass index (BMI) (*p* < 0.05).

**Conclusions:**

This study provides novel evidence of robust IFP adaptation to marathon running, characterized by increased FF and potential fluid shifts, suggesting enhanced cushioning and load dissipation. These findings highlight the importance of considering individual biomechanics in understanding IFP function and injury susceptibility. Future research should clarify the clinical implications of these adaptations for runner injury prevention and rehabilitation.

## Introduction

Marathon running, with its numerous health benefits, such as improved cardiovascular health, weight management, and mental well-being, has witnessed a steady increase in participation in recent years (Cai, Woods & Gao, 2021; Konwerski et al., 2021; Zheng et al., 2023). Despite the undeniable benefits of marathon running, long-distance running poses a heightened risk of sports injuries due to the repetitive loads and impacts exerted on the body. Among these injuries, knee injuries stand out as particularly prevalent (Zhang et al., 2021; Khan et al., 2022). In marathon running, the knee joint is required to endure high cumulative loading, with the infrapatellar fat pad (IFP) playing a crucial protective role situated between the joint capsule and synovium (Macchi et al., 2019; Nakano et al., 2004; Edama et al., 2022).

The IFP, also known as Hoffa’s fat pad, resides within a wedge-shaped space within the knee joint, specifically situated between the patella, anteroinferior femoral condyles, anterior superior margin of the tibia, and posterior to the patellar ligament (Nakano et al., 2004; Edama et al., 2022; Clockaerts et al., 2010). The IFP exhibits a complex anatomical structure with two distinct components: an inner portion composed of relatively firm, pillow-like adipose tissue and an outer portion consisting of softer adipose tissue that envelops the inner core (Nakano et al., 2004; Jiang, Fang & Wu, 2019). Composed primarily of white adipocytes, the IFP also contains adipose lobules and connective tissue, collectively contributing to its remarkable elasticity and compressibility, effectively absorbing and dispersing pressure generated during movement (Braun et al., 2022; Macchi et al., 2016; Stocco et al., 2021). Unlike visceral or subcutaneous fat, IFP adipocytes primarily serve protective and cushioning functions and exhibit resistance to metabolic influences (Diepold et al., 2015; Eymard et al., 2017). Furthermore, dense innervation of the IFP enables it to perceive pain, pressure, and temperature variations, thereby participating in the regulation of knee joint proprioception (Greif et al., 2020; Onuma et al., 2020). Moreover, the IFP contributes to synovial fluid production, facilitating joint lubrication, reducing friction, promoting even distribution of synovial fluid, nourishing the articular cartilage while maintaining its normal metabolism, ultimately ensuring smooth and flexible knee movement (Macchi et al., 2019; Edama et al., 2022; Macchi et al., 2018).

Abnormal or excessive biomechanical loads may induce morphological alterations, inflammation, fibrosis, and potential injury to the IFP, disrupting the knee joint’s normal function and causing pain and restricted mobility (Draghi et al., 2016). Although the IFP’s role in knee function has gained research attention, particularly in the context of osteoarthritis (Li et al., 2022; Tan et al., 2023; Wang et al., 2021), there is limited data on how it adapts to extreme athletic activities like marathon running, especially in healthy individuals.

Magnetic resonance imaging (MRI), a noninvasive high-resolution imaging modality, offers deep insights into the IFP’s morphology, size, and internal structure, enabling quantitative assessment of its tissue properties. This quantitative assessment is crucial for understanding IFP changes in various conditions (Hayashi et al., 2023). However, traditional quantitative MRI sequences like T1 mapping and T2 mapping require long scan times and variable conditions, making comparisons challenging. GE Healthcare’s magnetic resonance image compilation (MAGiC) is an advanced integrated sequence based on fast spin-echo imaging. Utilizing multiple inversion pulses and echo times within a single scan, MAGiC simultaneously acquires multiple contrast-weighted images and quantitative relaxation parameter maps (T1, T2, T1 fluid-attenuated inversion recovery (FLAIR), T2 FLAIR, short tau inversion recovery (STIR), and PD-weighted images) (Tanenbaum et al., 2017). This comprehensive acquisition is achieved by fitting the signal intensities acquired at various echo and inversion times to established mathematical models of tissue relaxation. By consolidating multiple acquisitions into a single scan, MAGiC significantly reduces overall scan time and enhances patient comfort. Emerging quantitative MRI techniques such as the iterative decomposition of water and fat with echo asymmetry and least-squares estimation quantitation (IDEAL-IQ) provide precise assessments of tissue lipidic content, which is valuable for characterizing tissue composition and detecting pathological changes. IDEAL-IQ utilizes multiple acquisitions with varying echo times to decompose the MRI signal into water and fat components based on their different chemical shifts. This allows calculation of the fat fraction (FF) as the ratio of the fat signal to the total signal (fat + water). IDEAL-IQ sequences yield FF, representing the relative amount of fat within the tissue, and the transverse relaxation rate (R2*). The R2* value, reflecting the tissue microenvironment, is derived from the decay of the MRI signal over time and is sensitive to magnetic field inhomogeneities.

This study evaluates changes in the IFP of amateur marathon runners before and after exercise using various quantitative MRI sequences. We comprehensively analyze microstructural and perfusion changes in the IFP to elucidate its role in knee joint health and inform injury prevention and rehabilitation strategies for runners.

## Materials and Methods

### Participants

This study was a prospective study, approved by the Ethics Committee of the First Hospital of Lanzhou University (LDYYLL2023-536) and all subjects signed the informed consent. Between May 20, 2024, and May 25, 2024, we recruited amateur marathon runners who had not undergone formal training or pursued marathon running professionally. Participants completed a detailed questionnaire to collect relevant demographic and health information.

Inclusion criteria were:

1. Completion of at least one formal marathon;

2. Regular running pattern (≥3 runs/week, ≥30 min/run, or ≥10 runs/month, ≥100 km/month);

3. No participation in marathons or equivalent training for two months prior to study enrollment;

4. No history of chronic diseases requiring long-term medication or previous knee trauma, surgery, or infection.

Exclusion criteria were:

1. Inability or unwillingness to undergo all required study scans;

2. Knee injury sustained during the study period;

3. Pre-existing knee injury evident on baseline MRI;

4. Any contraindication to MRI scanning;

A total of 14 amateur marathon runners (12 male, two female) meeting the specified criteria were enrolled in the study. All participants completed the full marathon distance at the 2024 Lanzhou marathon. Participants underwent an initial MRI scan within one week prior to the marathon. The second MRI scan was conducted within one week following the completion of the marathon. Participants were permitted to maintain their regular training routines, but were instructed to avoid participation in marathons or cross-country races during this period.

### MRI parameters

All MRI scans were performed using a 3.0T scanner (SIGNA Architect, GE, Boston, MA, USA) equipped with an eight-channel knee coil. Prior to each scan, participants rested in a seated position for one hour to ensure relaxation and minimize motion artifacts. During scanning, participants were positioned supine with their dominant knee in full extension within the knee coil. Sandbags and sponges were used to further stabilize the knee and minimize motion artifacts.

The following sequences were acquired for each participant:

Sagittal MAGiC: TR 4,000.0 ms, TE 19.1/95.7 ms, slice thickness 4.0 mm, interslice spacing 0.4 mm, field of view (FOV) 16 cm × 16 cm, matrix 256 × 256, bandwidth 31.25 kHz.

Axial T2-weighted fat-suppressed(T2-FS): TR 2,769.0 ms, TE 55.0 ms, slice thickness 4.0 mm, interslice spacing 0.4 mm, FOV 16 cm × 16 cm, matrix 260 × 260, bandwidth 31.25 kHz.

Sagittal PD-FS: TR 2,094.0 ms, TE 55.0 ms, slice thickness 4.0 mm, interslice spacing 0.4 mm, FOV 16 cm × 16 cm, matrix 260 × 260, bandwidth 31.25 kHz, inversion angle 111°.

Sagittal IDEAL-IQ: TR 9.9 ms, TE min, slice thickness 3.0 mm, FOV 28 cm × 28 cm, matrix 192 × 160, bandwidth 111.11 kHz.

### Infrapatellar fat pad quantitative MRI image analysis

All images were transferred to a dedicated post-processing workstation (GE SIGNA Architect 3.0 T MRI, MAGiC v100.1.1; GE, Boston, MA, USA). Two experienced radiologists (with over 7 and 3 years of experience in musculoskeletal radiology, respectively) independently analyzed the images using dedicated software. The radiologists were blinded to the participants’ identities and study time points.

Quantitative measurements were obtained as follows:

T1 and T2: Using the sagittal T1-weighted images from the MAGiC sequence, the radiologists identified the slice with the largest cross-sectional area of the IFP. This slice was used to determine the maximum IFP cross-sectional area. Regions of interest (ROIs) were manually drawn on this slice and the two adjacent slices (superior and inferior) to encompass the entire IFP. T1 and T2 values were then automatically calculated for each ROI.

FF and R2 *: The IDEAL-IQ sequence automatically generated R2* maps and FF maps. Using the FF map as a reference, the radiologists identified the slice with the largest IFP cross-sectional area and the two adjacent slices. ROIs were manually drawn on these slices to encompass the entire IFP, FF and R2* values were obtained.

FF represents the percentage of fat within a given voxel and is calculated based on the separated fat and water signal intensities:

$FF = \displaystyle{{{S_{fat}}} \over {{S_{fat}} + {S_{water}}}}$

S_fat:_ the fat signal component.

S_water:_ the water signal component.

The R2* reflecting the tissue microenvironment, is derived from the decay of the MR signal over time and is sensitive to magnetic field inhomogeneities.

${\rm R}2{\rm *} = - \displaystyle{{{\rm ln}\left( {{\rm S}\left( {{\rm TE}} \right)/{S_0}} \right)} \over {{\rm TE}}}$

TE: The echo time.

S(TE): This is the signal intensity at a specific TE, reflecting the signal decay due to relaxation and other effects.

S0: This is the initial signal intensity at TE = 0, representing the signal before any decay occurs.

**Figure 1:**
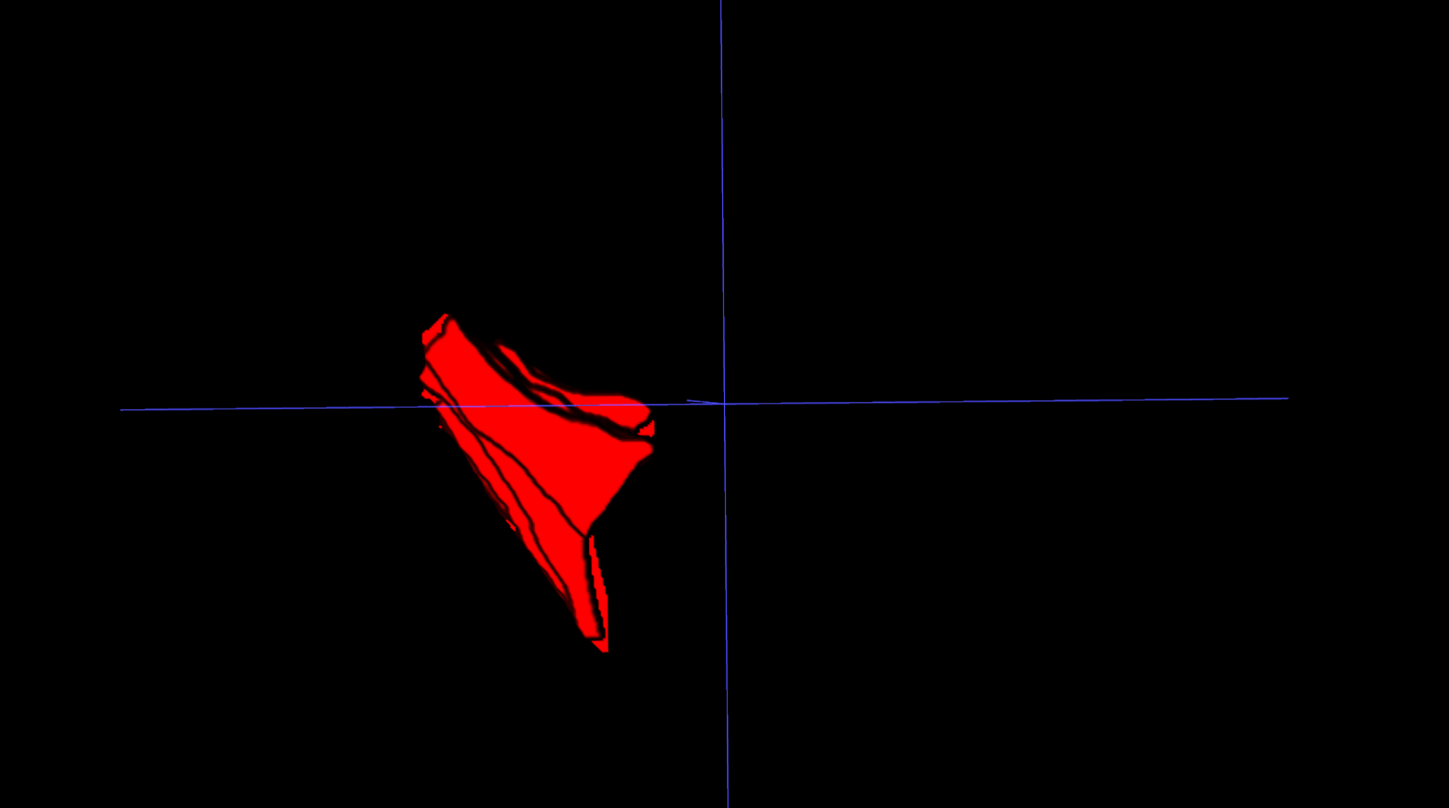
Three-dimensional visualization of the IFP. 3D reconstruction of the IFP, segmented from MRI data, illustrating its complex morphology and volume.

IFP volume: The IFP was manually segmented on PD-FS MRI images using 3D Slicer. Segmentation involved meticulous slice-by-slice delineation of the IFP margins to ensure accurate representation of IFP morphology. IFP volume was automatically calculated from the resulting 3D model (Fig. 1).

**Figure 2:**
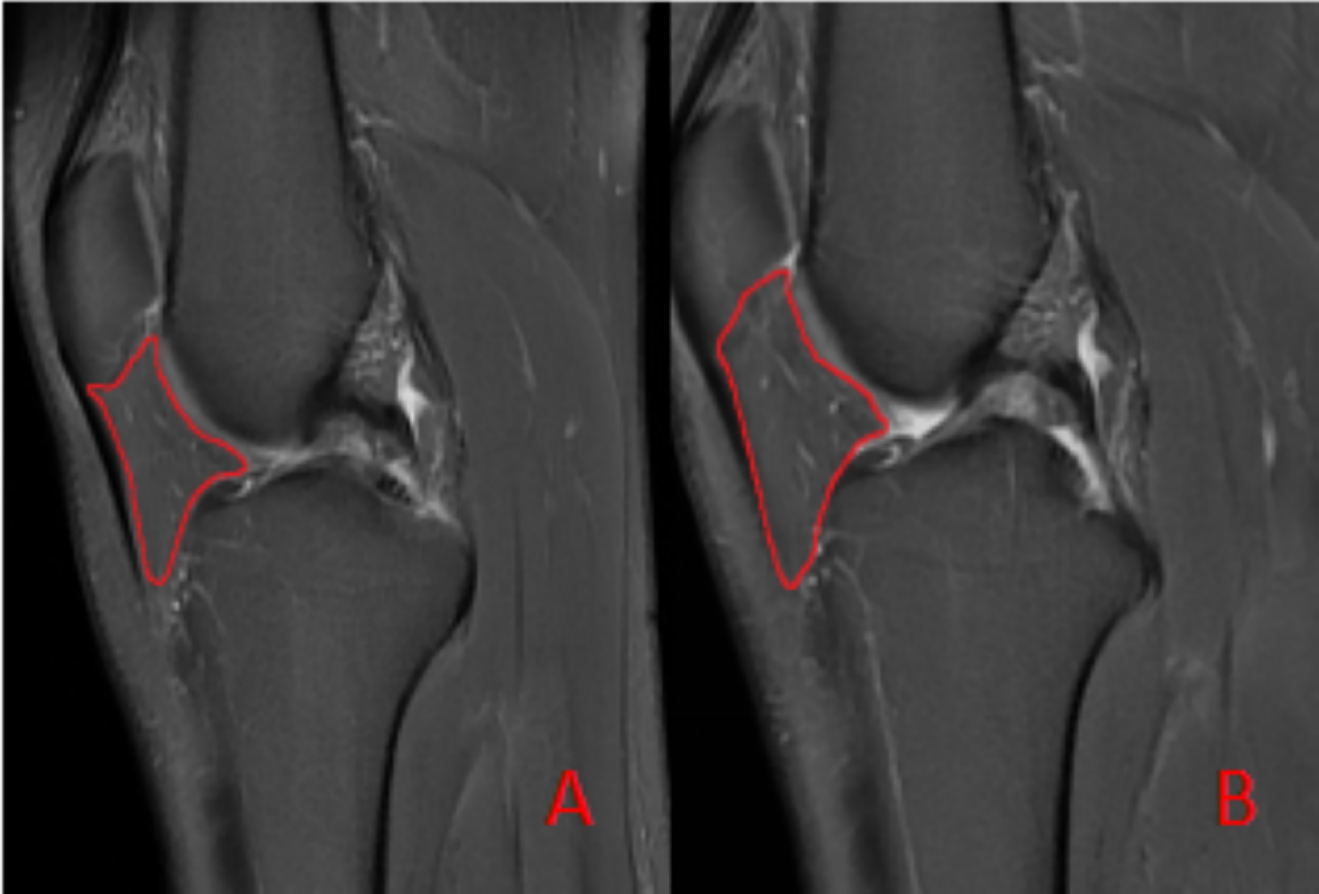
Schematic diagram illustrating the region of interest (ROI) delineation on PD-FS images. (A) Before the marathon, the maximum cross-sectional area of the IFP was 538 mm^2^. (B) After the marathon, the maximum cross-sectional area of the IFP was 507 mm^2^.

To ensure measurement accuracy and minimize error, standard deviation was controlled during ROIs delineation. To mitigate partial volume averaging artifacts, ROIs were carefully drawn to exclude vessels and the immediate vicinity, ensuring accurate representation of IFP composition (Fig. 2).

### Statistical analysis

Statistical analysis was performed using IBM SPSS version 25.0 (Armonk, NY, USA).Descriptive statistics for continuous variables, including age, height, weight, body mass index (BMI), T1, T2, FF, R2* values, IFP volume, and maximum cross-sectional area, were assessed for normality using the Shapiro-Wilk test. Variables exhibiting normal distribution are presented as mean ± standard deviation. Non-normally distributed variables are presented as median (interquartile range). Inter-observer reliability for quantitative MRI measurements was assessed using intraclass correlation coefficients (ICCs). Paired t-tests were used to compare pre- and post-marathon measurements of T1, T2, FF, R2* values, IFP volume and maximum cross-sectional area. Pearson correlation analysis was performed to assess the relationship between changes in quantitative MRI parameters and participant characteristics. A significance level of *p* < 0.05 was considered statistically significant for all analyses.

## Results

### Study participants

Of the 14 amateur marathon volunteers, 12 were male and 2 were female. Their ages ranged from 23 to 50 (37.14 ± 8.98) years, heights ranged from 165 to 186 (173.29 ± 7.13) cm, body weights ranged from 55 to 84 (68.29 ± 9.68) kg, BMIs ranged from 18.52 to 26.61 (22.69 ± 2.38) kg/m^2^, and the duration of their participation in the marathon ranged from 1 to 5 (3.0 ± 1.11) years (Table 1). All amateur marathoners did not show obvious symptoms such as knee injuries after running, and none of the morphologic imaging revealed any injury to the IFP after running.

**Table 1:** Participant characteristics.

Parameters	Mean ± SD	Range
Age (years)	37.14 ± 8.98	23–50
Height (cm)	173.29 ± 7.13	165–186
Weight (kg)	68.29 ± 9.68	55–84
BMI (kg/m^2^ )	22.69 ± 2.38	18.52–26.61
Previous marathon time (years)	3.0 ± 1.11	1–5

### Inter-reader variability and normality

The ICC values for MAGiC parameters were 0.78 (95% confidence interval (CI) [0.71–0.84]) for T1 and 0.86 (95% CI [0.82–0.91]) for T2. The ICC values for IDEAL-IQ were 0.95 (95% CI [0.93–0.96]) for FF and 0.87 (95% CI [0.82–0.90]) for R2*. The ICC value for the maximum IFP cross-sectional area was 0.76 (95% CI [0.52–0.88]). The ICC value for the IFP volume was 0.76 (95% CI [0.49–0.89]) (all *p* < 0.05). These ICC values indicate good to excellent interobserver agreement. Therefore, subsequent analyses were conducted using measurements obtained by the senior reader. Normality of data was assessed using the Shapiro-Wilk test. All variables were normally distributed.

### Quantitative MRI changes in the IFP

**Figure 3:**
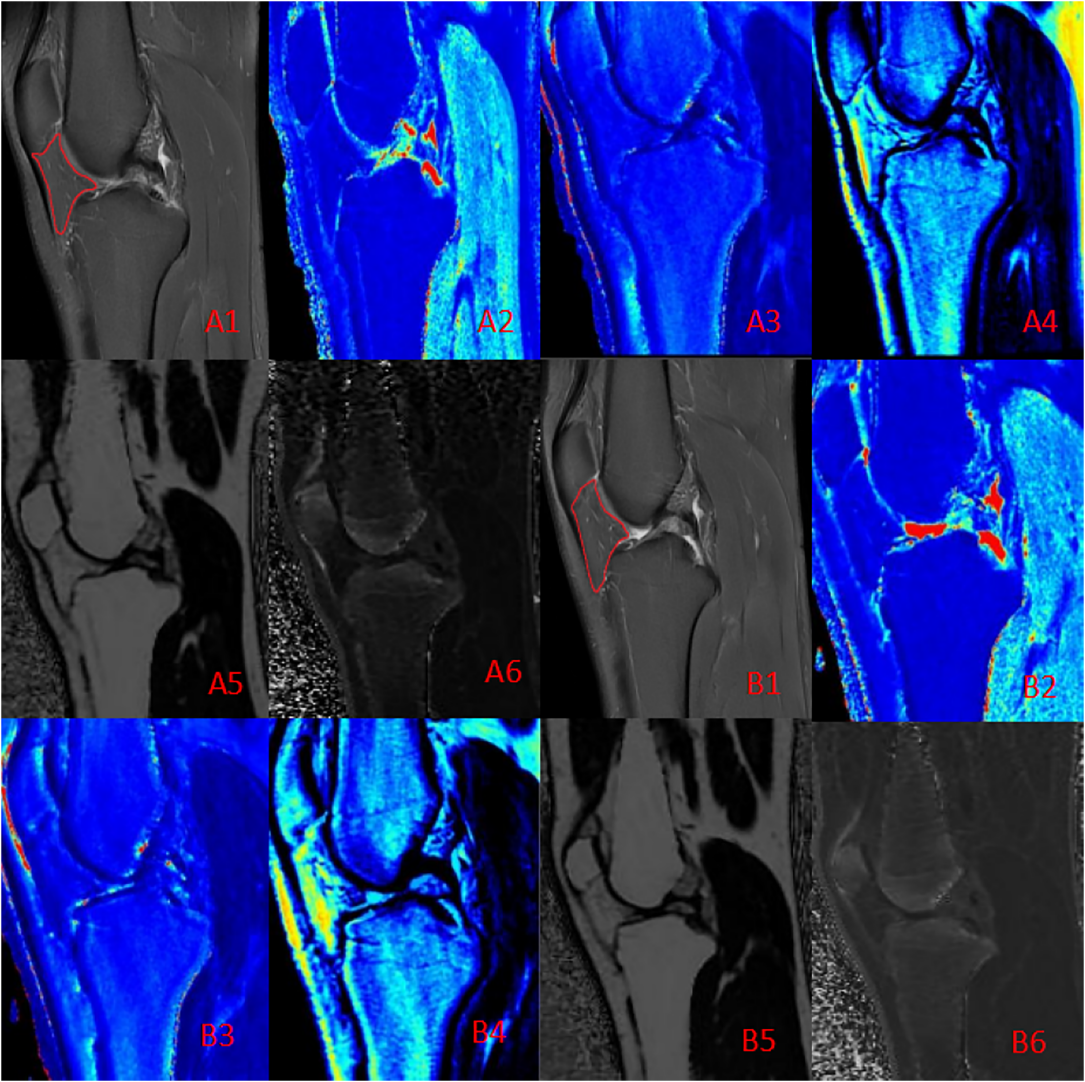
Quantitative maps of the IFP metrics from a male runner before (A) and after (B) the marathon. (A1, B1) PD-FS images; (A2, B2) T1 (pre: 457.50 ms, post: 451.76 ms); (A3, B3) T2 (pre: 48.21 ms, post: 44.42 ms); (A4, B4) PD (pre: 23.43, post: 22.34); (A5, B5) FF (pre: 80.12%, post: 80.88%); (A6, B6) R2* (pre: 59.62 Hz, post: 57.64 Hz). Post-marathon imaging reveals a slight decrease in T1 and T2 values, a increase in FF and a flatter shape than before the marathon.

All data were normally distributed, and paired t-tests were used to compare pre- and post-marathon differences in quantitative MRI parameters. After the marathon, the FF value of IFP was significantly higher than that before the marathon (*p* < 0.05), while IFP volume significantly decreased (*p* < 0.05). There was no significant change in T1 and T2 of IFP after marathon, but the overall trend was downward. Similarly, the maximum sagittal cross-sectional area of IFPs tended to decrease and adopt a flatter, more compact morphology following the marathon (Tables 2 and 3, Fig. 3).

**Table 2:** Comparison of IFP quantitative metrics before and after Marathon.

Metrics	Before marathon	After marathon	*p* value
T1 (ms)	501.53 ± 42.92	494.71 ± 37.21	0.27
T2 (ms)	53.93 ± 7.92	52.87 ± 5.32	0.25
Fat fraction (%)	76.51 ± 7.14	77.55 ± 7.42	<0.01
R2* (Hz)	71.07 ± 8.43	70.79 ± 9.13	0.66
Maximum cross-sectional area (mm^2^)	539.86 ± 97.50	518.64 ± 84.72	0.20
Volume (mm^3^)	21,394.11 ± 4,625.07	18,695.74 ± 3,273.89	<0.01

**Note:**

After the marathon, the FF value of IFP was significantly higher than that before the marathon (*p* < 0.05), while IFP volume significantly decreased (*p* < 0.05).

**Table 3:** Box plots illustrating changes in IFP parameters before and after the marathon.

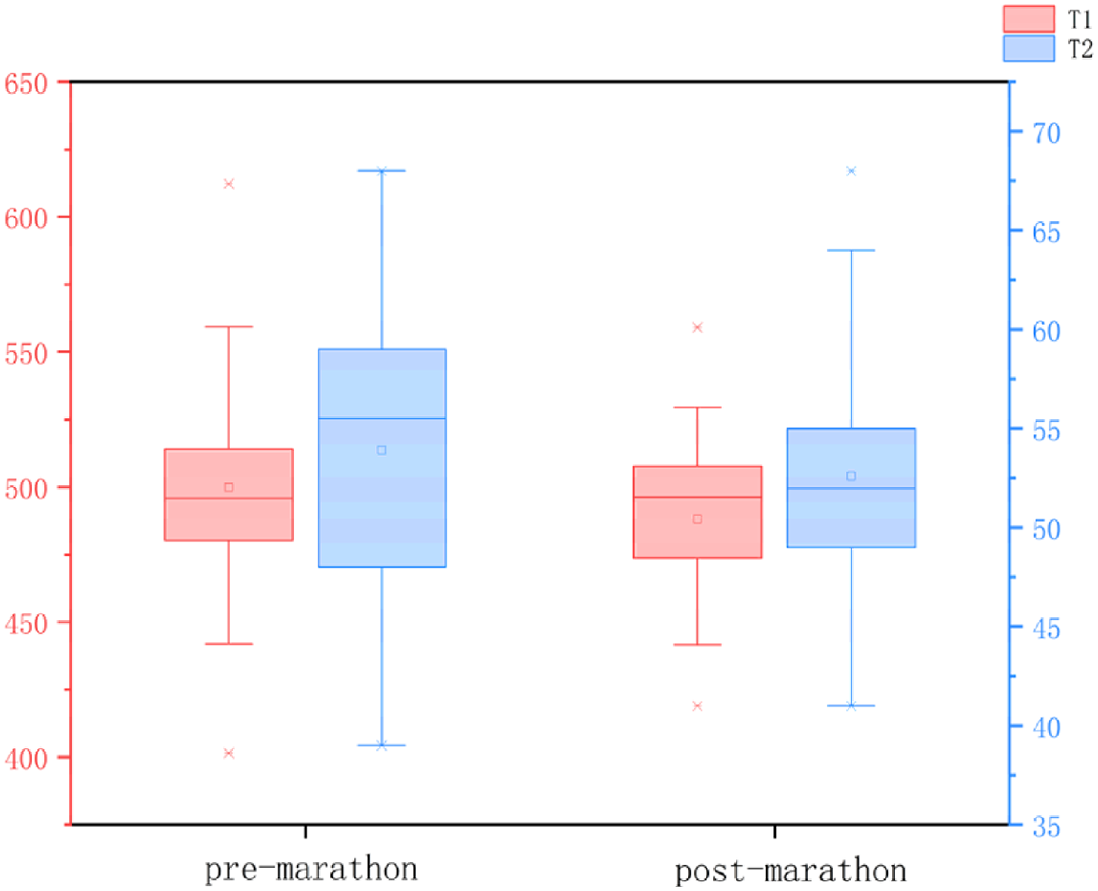	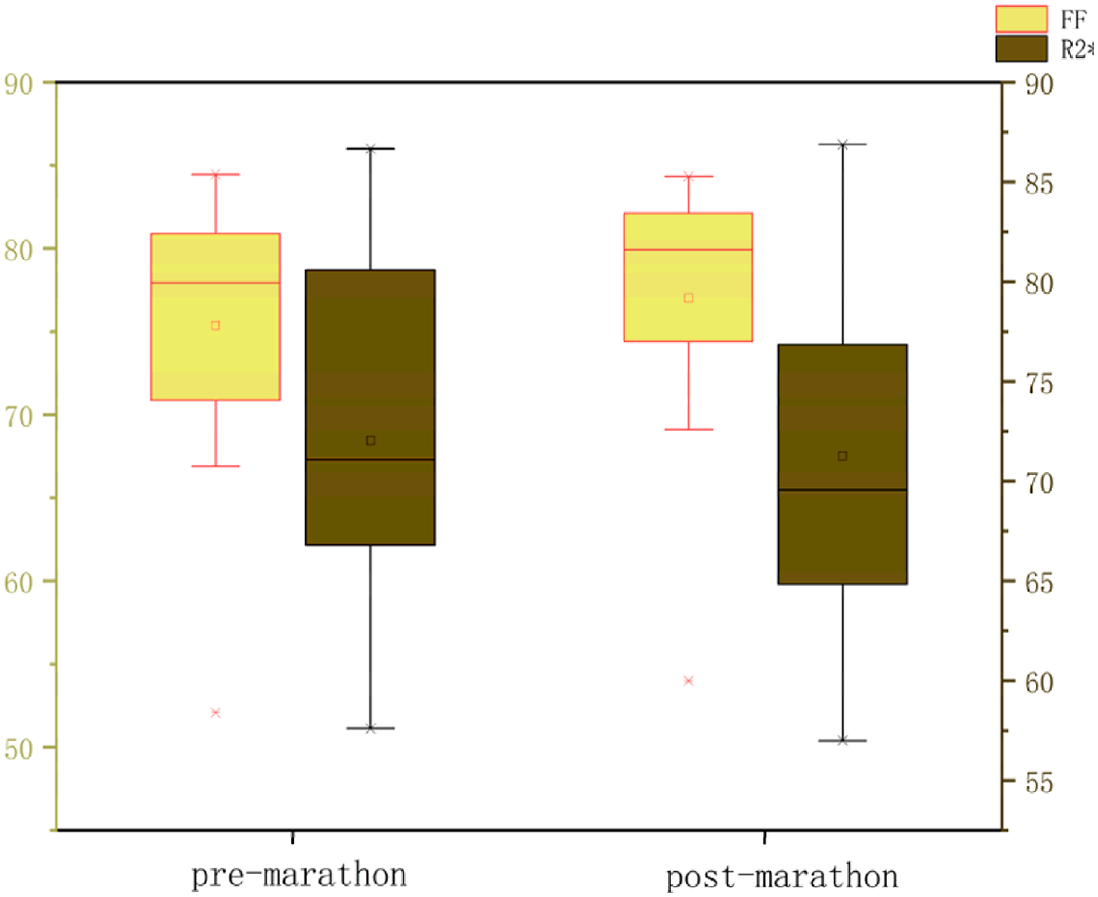	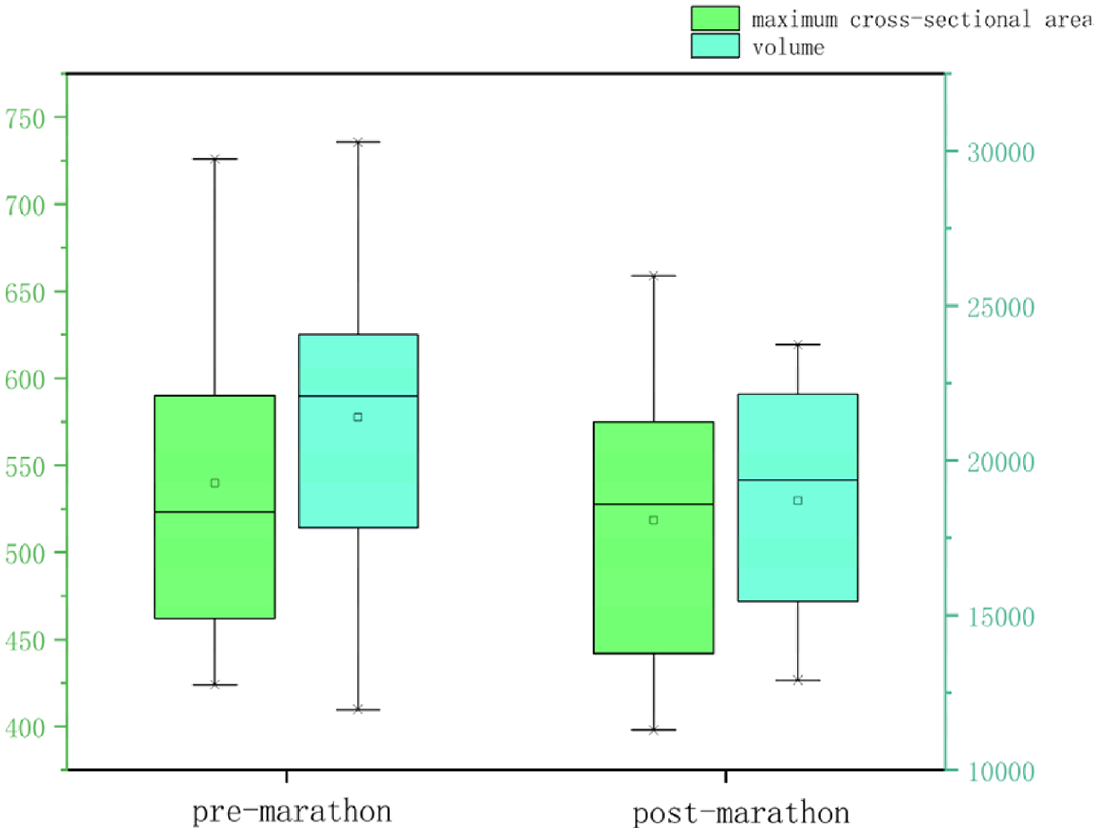

**Note:**

After the marathon, the FF value of IFP was significantly higher than that before the marathon (*p* < 0.05), while IFP volume significantly decreased (*p* < 0.05). There was no significant change in T1 and T2 of IFP after marathon, but the overall trend was downward.

### The relationship between changes in quantitative MRI parameters of the IFP and participant characteristics

Following the marathon, FF of the IFP was significantly elevated compared to pre-race measurements. Pearson correlation was used to analyze the correlation of participant characteristics and the ΔFF (Changes in FF values before and after the marathon) of the IFP. Results indicate that the age and height values had no correlation with the ΔFF of the IFP, but the weight and BMI were negatively correlated with the ΔFF of the IFP ( r =−0.64, r = −0.62) (Table 4).

**Table 4:** Correlations between fat fraction variation (ΔFF) and participant characteristics.

Metrics	Pearson correlation coefficient	*p* value
ΔFF and Age (y)	0.38	0.18
ΔFF and Height (cm)	−0.31	0.29
ΔFF and Weight (kg)	−0.64	0.01
ΔFF and BMI (kg/m^2^)	−0.62	0.02

**Notes:**

Pearson correlation was used to analyze the correlation of participant characteristics and the ΔFF (Changes in FF values before and after the marathon) of the IFP. Results indicate that the age and height values had no correlation with the ΔFF of the IFP, but the weight and BMI was negatively correlated with the ΔFF of the IFP (r = −0.64, r = −0.62).

## Discussion

This study provides one of the first quantitative assessments of IFP changes following marathon running, utilizing novel MAGiC and IDEAL-IQ imaging sequences, revealing a robust adaptive response of the interstitial compartment to acute exercise stress. The results indicated a significant increase (*p* < 0.05) in IFP FF following the marathon compared to the baseline measurement before the run. Although both T1 and T2 values exhibited a trend towards a decrease, these changes were not statistically significant. These findings demonstrate that marathon running may induce a significant increase in IFP adipose tissue proportion, potentially indicating a shift towards a more lipid-rich environment. This shift could reflect adaptations in metabolic pathways or microstructural changes within the interstitial compartment.

Moreover, analysis of the IFP before and after the marathon demonstrated a significant decrease in volume (*p* < 0.05) and a trend towards a decreased maximum cross-sectional area, demonstrating a more flattened or compact appearance following the run. This is likely attributable to the repetitive compressive forces endured during the marathon run. These findings provide novel insights into the biomechanical function of the IFP.

Our findings of altered IFP composition and morphology following a marathon provide novel insights into its adaptive response to intense exercise. The observed decrease in IFP volume aligns with the findings of Wallace, who reported IFP volume changes after anterior cruciate ligament reconstruction (Wallace et al., 2021). This suggests that IFP volume reduction may be a common response to various knee joint stressors, although the underlying mechanisms and long-term implications might differ. Similar volumetric changes in the IFP have also been observed in osteoarthritis (Fontanella et al., 2019). Furthermore, our results complement the work of Fontanella et al. (2022) who identified distinct IFP characteristics in osteoarthritis and post-traumatic lesions. While their study focused on pathological conditions, our research demonstrates that IFP adaptations also occur in response to physiological stress in healthy individuals. This highlights the dynamic nature of the IFP and its remarkable capacity for morphological and compositional change.

We propose that these immediate morphological and compositional changes are primarily driven by altered fluid dynamics rather than rapid adipocyte proliferation (Braun et al., 2022; Fontanella et al., 2017). The sustained high-intensity exertion of marathon running induces persistent quadriceps muscle contractions, exerting compressive forces on the IFP, with peak compression during knee extension. Simultaneously, the cyclical flexion and extension of the knee joint induce fluctuations in joint cavity pressure, further propelling fluid flow within the IFP and promoting its expulsion into adjacent tissues (Kakouris, Yener & Fong, 2021; Okita et al., 2020). Moreover, the intrinsic elastic deformation of the IFP itself, coupled with the compression of internal blood and lymphatic vessels under pressure, likely impedes fluid backflow, further contributing to this drainage effect (Clockaerts et al., 2010; Zhou et al., 2022). Consequently, due to the aforementioned pressure and fluid dynamic alterations, the adipose tissue percentage increases and the IFP reshapes following fluid drainage, as overall volume diminishes. This rapid adaptation may serve as a crucial cushioning mechanism, protecting the knee joint from repetitive high loads during long-distance running (Braun et al., 2022; Fontanella et al., 2017; Dragoo et al., 2010).

Researchers have recently turned their attention to the biomechanical function of the IFP. In an early study, Stevenson et al. (2006) utilized multi-angle 3D CT to analyze morphological changes in the IFP during knee flexion, highlighting its significant role as a mechanical cushion. Subsequently, Dragoo et al. (2010) utilizing dynamic MRI to observe changes in the IFP, revealed that weight-bearing significantly affects dynamic IFP changes during knee flexion and extension. Okita et al. (2020) utilized a three-dimensional MRI model to analyze the motion and volume changes of IFP during knee extension from 0° to 30° in healthy individuals. Results showed that the IFP significantly moved anteriorly and underwent volume changes, which were closely related to tibial external rotation and patellar lateral displacement (Okita et al., 2020). While previous studies primarily focused on the shape and volume changes of the IFP during knee motion, this study pioneers a novel quantification of the IFP’s mechanical function through MRI-based compositional analysis. This study challenges the traditional view of the IFP as a passive cushion, revealing it to be a dynamic and complex structure. Our findings demonstrate that the IFP’s shape and composition adapt in response to varying exercise loads, suggesting a potential role in maintaining knee joint stability. Future research should explore IFP fluid flow mechanisms, alterations in mechanical properties, and its intricate interactions with surrounding tissues to comprehensively elucidate its biomechanical function. The IFP plays a crucial role in knee osteoarthritis (KOA) development, with studies revealing MRI signal intensity, morphology, and volume changes in IFP of KOA patients linked to disease severity and prognosis (Fontanella et al., 2019, 2022; Cen et al., 2023). While our study focused on healthy runners, the observed decrease in IFP T1 and T2 values following a marathon raises the intriguing possibility that moderate exercise might influence IFP composition and potentially inflammation in conditions such as osteoarthritis. Given that elevated IFP signal intensity, often indicative of inflammation, is frequently observed in KOA, the opposite effect seen in our healthy cohort warrants further investigation.

Further analysis demonstrated a significant negative correlation between the change in FF and both body weight and BMI, indicating that higher body weight is a key determinant of IFP biomechanical properties. This finding is consistent with Dragoo et al.’s (2010) observation, using dynamic MRI, that IFP morphological adaptation is reduced during weight-bearing knee joint exercise compared to non-weight-bearing exercise. This convergence of findings from different methodologies underscores the significant role of body weight in modulating the mechanical stimuli experienced by the IFP and its subsequent adaptation (Ricatti et al., 2021). This suggests that individuals with higher body weight, experiencing prolonged exposure to greater joint loading, may have developed structural adaptations in their IFP that reduce its sensitivity to exercise-induced biomechanical alterations. Future research should investigate the relationship between IFP organization, body weight, and weight-bearing adaptations to elucidate the specific biomechanical response mechanisms of IFP during exercise.

The increasing prevalence of total knee arthroplasties (TKAs) has fueled debate regarding the optimal intraoperative management of the IFP, particularly concerning its preservation or resection. While some studies suggest that resecting the IFP may provide short-term pain relief, a growing body of evidence indicates that such resection can potentially exacerbate postoperative pain, increase the risk of compromised blood flow to the patellar tendon, and ultimately lead to patellar tendon scar formation and shortening, thereby hindering knee mobility (Jiang, Fang & Wu, 2019; Chougule et al., 2015; Belluzzi et al., 2019). Our findings, together with existing literature, highlight the pivotal role of the IFP in maintaining knee joint homeostasis and warrant cautious consideration of the potential risks associated with IFP removal during clinical decision-making.

The high interobserver reliability (ICCs ranging from 0.76 to 0.95, all *p* < 0.05) supports the robustness and reproducibility of our quantitative MRI measurements. This excellent agreement between observers strengthens the validity of our findings, indicating that the observed IFP changes are not due to variations in measurement technique. This level of reliability is particularly important for detecting subtle changes in IFP morphology and composition, further highlighting the potential of quantitative MRI as a valuable tool for assessing early IFP changes in athletes. Prior ultrasound studies have revealed a unique bilayered structure within the IFP, comprising superficial and deep layers (Vera-Pérez et al., 2017). The superficial layer, adjacent to the patellar ligament, is characterized by larger, loosely arranged fat lobules, while the deeper layer, situated closer to the bone, exhibits smaller, tightly packed fat lobules. During knee flexion, the superficial layer experiences greater pressure, leading to a significant reduction in the size and thickness of its fat lobules, whereas the deep layer remains largely unaffected. These observations, including the distinct responses of superficial and deep layers to knee flexion and their unique blood supply patterns during joint movement (Nakanishi et al., 2021), highlight significant disparities in stress distribution and adaptation mechanisms within the IFP. Future research could investigate subdividing the IFP into subregions based on force directions, facilitating a more comprehensive analysis of its structure and function.

This study employed quantitative MRI techniques to investigate the effects of marathon running on IFP morphology and composition in amateur athletes, offering novel insights into the adaptive responses of this tissue to strenuous exercise. However, several limitations merit acknowledgement. The relatively small sample size and lack of a control group constrain the generalizability of our findings and preclude definitive causal inferences regarding IFP adaptations. Additionally, the cross-sectional design, capturing IFP characteristics at a single post-marathon time point, limits our ability to establish a clear temporal profile of these adaptations. Future investigations employing longitudinal cohort designs with long-term follow-up are warranted to address these limitations. Methodologically, our reliance on morphological and compositional analyses provides an indirect assessment of IFP mechanical properties. While manual ROI segmentation may introduce subjectivity and impact measurement accuracy, future studies could incorporate biomechanical testing or finite element analysis to directly quantify functional parameters such as stress-strain relationships and energy absorption. Moreover, exploring more sophisticated and automated segmentation techniques could enhance measurement precision. Finally, this study primarily provides observational data, leaving the specific molecular mechanisms underlying marathon-induced IFP adaptations largely unexplored. Future research incorporating animal models or cellular experiments could elucidate these mechanisms, focusing on alterations in fluid flow, cellular metabolism, and tissue remodeling at the gene expression, protein synthesis, and cellular signaling pathway levels. Such investigations hold promise for informing the development of more effective strategies for preventing and treating sports-related injuries.

## Conclusion

This study reveals a significant association between long-distance marathon running and increased IFP FF, accompanied by a flatter and more compact morphology. This observation suggests that acute exercise loading may induce fluid extrusion and drainage from the IFP, potentially representing an adaptive response. However, the precise mechanism underlying IFP fluid flow and its implications for joint health remain to be elucidated. Further investigation is warranted to delineate the biomechanical mechanisms driving IFP fluid dynamics and to evaluate its relationship with articular cartilage metabolism, inflammatory responses, and injury risk. This line of research holds promise for informing the development of evidence-based strategies for sports injury prevention and rehabilitation, ultimately contributing to optimizing athletic training regimens and enhancing joint health.

## Supplemental Information

10.7717/peerj.19123/supp-1 Supplemental Information 1 All volunteer raw data. The changes in MR quantitative parameters of the infrapatellar fat pad before and after the volunteer marathon

## Additional Information and Declarations

### Data Availability

The following information was supplied regarding data availability:

The raw measurements are available in the Supplemental Files.
